# Black Phosphorus Tagged Responsive Strontium Hydrogel Particles for Bone Defect Repair

**DOI:** 10.1002/advs.202408284

**Published:** 2024-11-06

**Authors:** Zhengwei Liu, Hui Zhang, Jingjing Gan, Yuanjin Zhao, Yongxiang Wang

**Affiliations:** ^1^ Department of Orthopedics Northern Jiangsu People's Hospital Clinical Teaching Hospital of Medical School Nanjing University Yangzhou 225001 China; ^2^ Department of Rheumatology and Immunology Nanjing Drum Tower Hospital School of Biological Science and Medical Engineering Southeast University Nanjing 210096 China; ^3^ Shenzhen Research Institute Southeast University Shenzhen 518071 China; ^4^ Department of Orthopedics Northern Jiangsu People's Hospital Yangzhou 225001 China

**Keywords:** black phosphorus, bone defect repair, hydrogel, microparticle, responsive, strontium

## Abstract

Hydrogel‐derived implants have proven value in bone tissue regeneration, and current efforts have concentrated on devising strategies for producing functional implants with desired structures and functions to improve therapeutic outcomes. Herein, a novel black phosphorus (BP) tagged responsive strontium (Sr) hydrogel particles are presented for bone defect repair. By applying microfluidic technology, Sr and carboxymethyl chitosan, and BP are integrated into poly(N‐isopropyl acrylamide) (pNIPAM) hydrogel matrix to generate such microparticles called pNBCSMs. Upon exposure to near‐infrared irradiation, the pNBCSMs experience volume shrinkage and provoke the extrusion of the incorporated Sr, ascribed to the photothermal conversion ability of BP and the thermosensitivity of pNIPAM. In vitro and in vivo experimental results reveal that pNBCSMs subjected to near‐infrared light display superior anti‐inflammatory, anti‐apoptotic, bacterial inhibitory, as well as osteogenesis‐promoting effects, thereby effectively improving defective cranial bone repair. These features suggest that the proposed pNBCSMs can be promising candidates for bone repair.

## Graphical abstract

A type of black phosphorus (BP) tagged responsive strontium (Sr) hydrogel particles (pNBCSMs) is presented for bone defect repair. The developed pNBCSMs demonstrate controllable drug release, superior biocompatibility, powerful bone repair effects, and potential personalized treatments, which breaks through the limitations of traditional hydrogel biomaterials and positioning themselves as a valuable addition to the clinical applications.

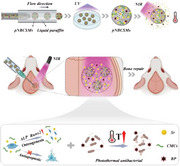

## 1 Introduction

Bone defects caused by trauma, tumors, or osteoarthritis are prevalent in clinical orthopedic diseases.^[^ ^1^, ^2^, ^3^ ^]^ Traditional approach to treating bone defects involves the placement of various implants such as biological bone, synthetic bone grafts, non‐metallic materials, or metallic materials.^[^ ^4^, ^5^, ^6^, ^7^ ^]^ Although with much effectiveness, these strategies are usually limited by donor deficiency, inflammatory response, and integration failure.^[^ ^8^, ^9^ ^]^ To address these challenges, hydrogel‐based implants with bioactive properties have been employed to promote bone tissue regeneration.^[^ ^10^, ^11^, ^12^ ^]^ Especially, multiple functional elements have been incorporated into hydrogel matrices to enhance their therapeutic effects via slow and controlled release.^[^ ^8^, ^13^, ^14^, ^15^, ^16^ ^]^ Despite advanced progress in bone repair, most research on hydrogel implants has focused on organic components such as polysaccharides and proteins while neglecting the importance of trace elements in bone regeneration.^[^ ^17^, ^18^, ^19^ ^]^ In addition, conventional hydrogel implants largely impede drug penetration to the defected area and form a certain physical obstacle to new tissue growth due to their large volume.^[^ ^20^, ^21^, ^22^ ^]^ Thus, a new hydrogel‐based implant with the desired properties is still anticipated for bone repair.

**Figure 1:**
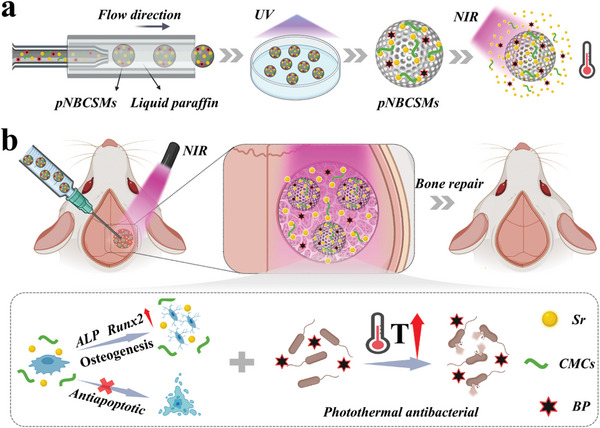
Schematic diagram of pNBCSMs for bone defect repair a) Schematic of the preparation process of pNBCSMs. b) Responsive pNBCSMs promote bone defect repair under NIR irradiation after injection administration and its mechanism of action.

In this paper, we present a novel black phosphorus (BP) tagged responsive strontium (Sr) hydrogel particles for bone defect repair (**Figure** **1**). Sr is an essential trace element inhibiting bone resorption and increasing bone formation, and thus holds growing promise for bone tissue engineering.^[^ ^23^, ^24^ ^]^ In contrast, as an appealing 2D‐layered nanomaterial, BP exhibits satisfactory biocompatibility and unique photothermal conversion properties and can promote bone regeneration and reconstruction by stimulating calcium absorption.^[^ ^25^, ^26^, ^27^, ^28^ ^]^ Particularly, BP has been utilized as a photothermal agent in conjunction with temperature‐responsive materials like poly (N‐isopropyl acrylamide) (pNIPAM) for intelligent drug delivery and tissue regeneration.^[^ ^29^, ^30^, ^31^, ^32^ ^]^ Besides, hydrogel particles with spherical shapes ensure their injectability and facilitate drug penetration to the lesion through gaps between the particles.^[^ ^33^ ^]^ Significantly, particles generated by microfluidics are known for their precise and adjustable size, enabling their extensive development in drug delivery, medical diagnosis,^[^ ^34^, ^35^ ^]^ and cell encapsulation.^[^ ^36^, ^37^, ^38^, ^39^, ^40^, ^41^, ^42^ ^]^ Therefore, we conceive that integrating Sr and BP into a pNIPAM hydrogel matrix using microfluidic technology is expected to create a new type of hydrogel microparticles for bone repair.

Here, we developed the desired pNIPAM hybrid hydrogel microparticles (pNBCSMs) containing Sr, carboxymethyl chitosan (CMCs), and BP utilizing a microfluidic platform for photothermally responsive release of Sr for bone regeneration. By adjusting the concentration of the incorporated CMCs component, multiple physicochemical properties of the hybrid hydrogel were optimized, including mechanical strength, swelling performance, degradation rate, as well as porous structures. Besides, the dimensions of pNBCSMs could be resized by controlling the outer and inner flow rates, making them adaptive to various practical application requirements. Taking advantage of the photothermal conversion capability of BP and thermal sensitivity of pNIPAM hydrogel, the loaded Sr could be responsively released from the resulting pNBCSMs upon exposure to near‐infrared (NIR) light. In vitro experiments demonstrated that the pNBCSMs had osteogenic capacity and displayed anti‐inflammatory, anti‐apoptotic, and antibacterial effects. Moreover, based on constructing cranial defect models, we have confirmed that pNBCSMs subjected to NIR light effectively accelerated bone defect repair in vivo. These results indicate that the designed pNBCSMs hold significant application potential for bone regeneration.

## 2 Results and Discussion

In a typical experiment, the substrate materials of pNBCSMs were first optimized by adjusting the CMCs concentration. In detail, the pNIPAM, N,N″‐Methylenebisacrylamide (BIS), and BP were dissolved and mixed to obtain the pNIPAM/BP (NP/BP) hydrogel precursor solution. Various contents of CMCs were added to the NP/BP hydrogel precursor solution, and NP/BP/CMCs hydrogel was obtained after ultraviolet (UV) irradiation. Following that, the NP/BP/CMCs hydrogel was immersed in SrCl_2_ solution to generate NP/BP/CMCs@Sr hydrogel. The elasticity and compression resistance of the material are crucial for bone tissue defect repair, so we explored the mechanical properties of the above composite hydrogels. The rheological results visualize that the values of the loss modulus (G″) are generally below the storage modulus (G′) for all tested samples except for the NP/BP/1.0% CMCs hydrogel, indicating the formation of the hydrogel networks, which was consistent with the results shown in Figures S1 and S2 (Supporting Information). Notably, the G′ and G″ of NP/BP/CMCs@Sr were generally higher than those of NP/BP/CMCs hydrogels. This might be attributed to the complexation of CMCs by Sr ion, which enhanced the cross‐linking within the composite hydrogels. In addition, the composite hydrogels containing 0.5% CMCs exhibited the highest G′ and G″ due to excess CMCs hindering the cross‐linking of the hydrogels, thus reducing their mechanical strength. Additionally, the maximum axial compression force of the NP/BP/CMCs@Sr hydrogels was enhanced compared to that of the NP/BP/CMCs hydrogels, wherein NP/BP/0.5% CMCs@Sr hydrogels had the strongest mechanical properties, which was consistent with the rheological results (Figure S3, Supporting Information). Furthermore, scanning electron microscopy (SEM) result visualized that NP/BP/CMCs@Sr hydrogels displayed porous structures that were beneficial for cell outgrowth and substance exchange (Figure S4, Supporting Information). Of note, the internal microstructures of the NP/BP/CMCs@Sr hydrogels were denser than those of the NP and NP/BP/CMCs hydrogels, which further explained that the mechanical strength of NP/BP/CMCs@Sr hydrogels was significantly improved. These results suggest that NP/BP/0.5% CMCs@Sr hydrogels have good mechanical properties, which fulfills the requirements of biomaterials for filling bone defects.

Previous studies have demonstrated that hydrophilicity on the material surface not only suppresses inflammation but also improves its biocompatibility.^[^ ^43^ ^]^ Therefore, we conducted water contact angle (WCA) experiments to evaluate the hydrophilicity of NP/BP/CMCs@Sr hydrogels. As shown in Figure S5 (Supporting Information), the WCA of NP/BP/CMCs@Sr hydrogels and NP/BP/CMCs hydrogels gradually decreased with the addition of CMCs, indicating an increase in hydrophilicity. This property could be ascribed to abundant hydroxyl and carboxyl groups within CMCs, a polysaccharide compound.^[^ ^44^ ^]^ Considering the mechanical and biological properties of the composite hydrogels, we chose 0.5% as the final concentration of CMCs for subsequent experiments.

**Figure 2:**
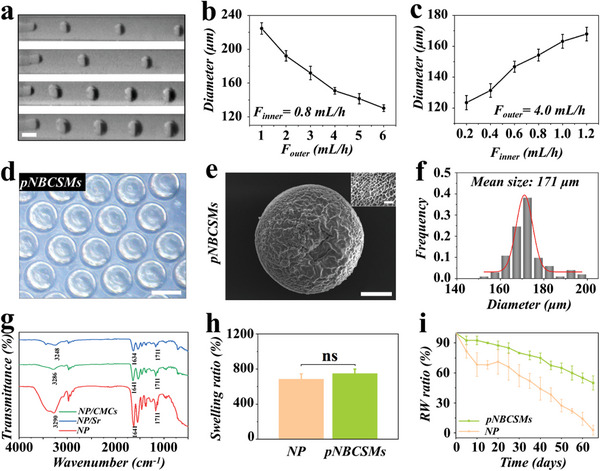
Preparation and characterization of the pNBCSMs a) Real‐time generation image of droplets in the microfluidic device with different flow rates of inner and outer phases. The scale bar is 200 µm. b,c) Relationships of microsphere diameters with the outer flow rate (b) and the inner flow rate (c) (*n* = 50 for each group). d) Optical microscopy image of pNBCSMs. The scale bar is 200 µm. e) SEM images of the whole view and surface view of pNBCSMs. The scale bars are 50 and 10 µm (inset). f) Statistical size distribution of pNBCSMs. g) FTIR spectra of hydrogels. h) Swelling ratio of hydrogels (pH 7.4, 37 °C). i) Remaining weight (RW) ratio of hydrogels (pH 7.4 at 37 °C).

Monodisperse pNBCSMs were then prepared by utilizing microfluidic technology. Within a microfluidic device, the hydrogel precursor solution (inner phase) was cut into individual droplets by the shear force of liquid paraffin (outer phase), and pNBCSMs were generated through UV irradiation. Particularly, pNBCSMs of different sizes could be prepared by adjusting the rates of inner and outer phases (**Figure** **2**a–c). It was observed that the diameter of pNBCSM decreased with the increase in the outer phase rate but increased with the inner phase rate. The obtained pNBCSMs displayed spherical shape, porous microstructures, as well as excellent monodispersity within the range of 160–185 µm (Figure 2d–f). we use microfluidic technology to prepare microparticulate hydrogels, which increases the specific surface area of the hydrogel and facilitates drug and nutrient penetration, as well as optimizes the bone regeneration environment by reducing the implant's physical obstruction of the nascent tissue. According to the energy dispersive spectrometer (EDS) analysis, the presence of the Sr element and P element in pNBCSMs indicated successful incorporation of Sr within the pNBCSMs, and are uniformly distributed in pNBCSMs (Figure S6, Supporting Information). In addition, high‐resolution TEM (HRTEM) images showed that the BP we used showed highly concentrated crystal streaks, which ensured the bioactivity of BP (Figure S7, Supporting Information). Fourier transform infrared (FTIR) spectra of NP, NP/Sr and NP/CMCs hydrogel were also provided to verify the construction of pNBCSMs (Figure 2g). The peaks at 3290 and 1641 cm^−1^ attributed to the hydroxyl group and amide group in NP, respectively, which shifted to a lower wavenumber in NP/Sr hydrogel due to the involvement of the hydroxyl group and amide group in the coordination of Sr. However, compared to the spectrum of NP hydrogel, the absorption bands of the OH group and amide group in NP/CMCs hydrogel had no significant polarization, which implied that CMCs may be physically bound to hydrogel particles. Furthermore, we investigated the swelling and degradation properties of pNBCSMs. As shown in Figure 2h, the swelling ratio of pNBCSMs was found to be similar to that of NP hydrogel, reaching approximately eight times its initial state. It is widely accepted that hydrogels with a high swelling ratio can absorb tissue‐inflammatory exudates and maintain a moist environment for tissue regeneration, which can facilitate bone tissue defect repair.^[^ ^45^ ^]^ As depicted in Figure 2i, NP hydrogel degraded essentially completely in less than two months, while pNBCSMs retained ≈50% of their weight after two months, which was desirable for in vivo bone tissue regeneration applications. Hydrogel degradation and bone repair showed a dynamic equilibrium within 2 months. The result of hydrogel degradation rate show that in the early stage of bone repair, with the degradation of pNBCSMs, the emerging bone tissue effectively fills and remodels the bone defect area, which is sufficient to validate the feasibility of this material.

**Figure 3:**
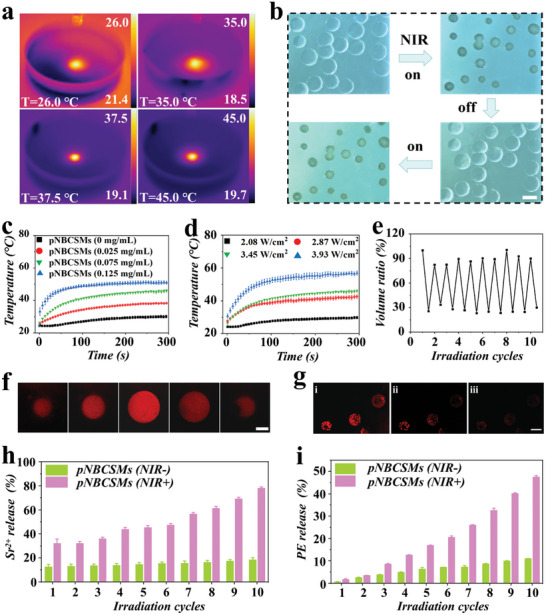
Characterization of the pNBCSMs a,b) The relationship between temperature of pNBCSMs and BP concentration (a) and NIR power (b) (*n* = 3 for each group). c) Thermal images of pNBCSMs after NIR irradiation. d) Optical images of pNBCSMs under NIR on and off. The scale bar represents 200 µm. e) The volume ratio of pNBCSMs under 10 cycles under NIR on and off. f) The layer‐by‐layer photographs of pNBCSMs containing rhodamine B obtained from CLSM. The scale bar represents 100 µm. g) The fluorescence photographs of pNBCSMs containing PE before (i) and after one (ii) and two (iii) NIR cycles. The scale bar represents 200 µm. h,i) The cumulative release percentage of Sr^2+^ (h) and PE (i) from pNBCSMs with or without NIR irradiation (*n* = 3 for each group).

It is well known that the volume of pNIPAM hydrogel exhibits abrupt changes in form when the temperature is above its lower critical solution temperature (LCST) ≈32 °C.^[^ ^36^, ^46^ ^]^ We explored the LCST of pNBCSMs containing CMCs. As recorded in Figure S8 (Supporting Information), the LCST of pNBCSMs increased as the concentration of CMCs increased, and the LCST of pNBCSMs (0.5% CMCs) was ≈40 °C, thus offering a suitable LCST for in vivo drug delivery applications. Besides, BP, with high photothermal conversion efficiency, endowed the pNBCSMs with the ability to generate heat under NIR light exposure (**Figure** **3**a). Particularly, the temperature‐increasing rate of pNBCSMs increased with BP concentration and NIR light power (Figure 3c,d). Considering the negative effect of high temperature on the normal tissues and cells, 0.075 mg mL^−1^ BP and 3.45 W cm^−2^ of NIR power were decided as the optimized parameters for subsequent experiments, under which condition the temperature of pNBCSMs reached ≈45 °C after 300 s of irradiation. Besides, after being irradiated with NIR light, the pNBCSMs underwent a significant shrinkage and returned to the initial morphology when turning off NIR light (Figure 3b). Within 10 cycles of NIR irradiation, pNBCSMs underwent contraction and recovery repeatedly, demonstrating their reproducible temperature responsiveness (Figure 3e).

In order to observe the distribution of Sr within pNBCSMs, we incorporated rhodamine‐B with the inherent red fluorescent signal as a substitute into pNBCSMs and obtained layer‐by‐layer fluorescence scanning images. As displayed in Figure 3f and Figure S8c (Supporting Information), rhodamine‐B was uniformly distributed in pNBCSMs. To further evaluate the release behavior of polysaccharide molecules from pNBCSMs under NIR triggering, we encapsulated the substitute phycoerythrin (PE) with an intrinsic fluorescence signal in pNBCSMs. It was observed that the red fluorescence signal of pNBCSMs gradually decreased after two times NIR irradiation (Figure 3g), which indicated that PE could be controllably released from pNBCSMs under NIR triggering. In addition, the cumulative release amount of Sr^2+^ and PE increased with NIR irradiation times, reaching 78.2% and 46.2% after 10 cycles, separately. In contrast, the cumulative release amount of Sr^2+^ and PE from pNBCSMs was only ≈18.6% and 10% without NIR irradiation, separately (Figure 3h,i). This result revealed that the release process of drugs from pNBCSMs could be effectively controlled by NIR irradiation. Thus, our proposed pNBCSMs system can achieve precise control of Sr release in time and space through photothermal conversion to enhance the targeting and efficiency of therapeutic effects.

Biocompatibility of biomaterials is crucial for tissue repair in vivo,^[^ ^15^, ^47^, ^48^ ^]^ so Mouse Embryonic Osteoblasts (MC3T3‐E1) cells were co‐cultured with pNBCSMs, and Live/dead staining and Cell Counting Kit‐8 analysis were conducted to assess the biocompatibility of prepared pNBCSMs. As shown in Figure S9a (Supporting Information), there were few dead cells in all groups, revealing low cell toxicity of pNBCSMs. However, cell growth was significantly accelerated in the pNBCSMs group on day 3, which could be attributed to the incorporation of Sr and CMCs (Figure S9b,c, Supporting Information). Furthermore, the apoptosis rate of the MC3T3‐E1 cell in the pNBCSMs group was 0.86%, lower than in the other four groups (Figure S9d–i, Supporting Information). This result indicates that pNBCSMs possessed a positive anti‐apoptotic effect on MC3T3‐E1 cells, thus contributing to tissue regeneration.

**Figure 4:**
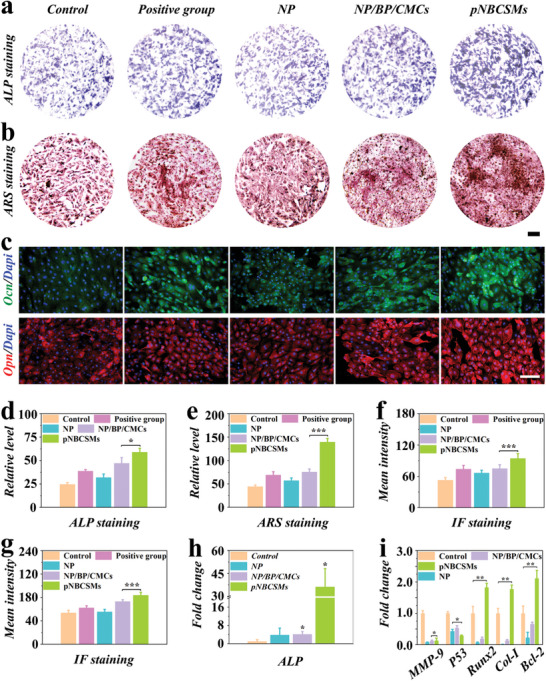
Pro‐osteogenesis effect of pNBCSMs a,b) Representative images of ALP staining (a) and ARS staining (b) in each group. The scale bar is 25 µm. c) Representative IF staining images of Ocn and Opn on MC3T3‐E1 cells with different treatments at day 7. d) Quantitative analysis of the ALP staining (n = 3). (e) Quantitative analysis of ARS (*n* = 3). f,g) Statistical analysis of Ocn (f) and Opn (g) IF staining on MC3T3‐E1 cells based on IF staining images (*n* = 3). The scale bar represents 80 µm. h,i) Real‐time Quantitative PCR (RT‐qPCR) analysis on related gene expression in MC3T3‐E1 cells in each group. *p < 0.05, **p < 0.01, and ***p < 0.001, one‐way ANOVA using the Tukey post‐hoc test.

The process of bone formation includes osteoblast differentiation and bone mineralization. Alkaline phosphatase (ALP) and bone mineralization nodule (BMN) expression levels are widely used to evaluate the capacity of bone formation.^[^ ^49^ ^]^ As exhibited in **Figure** **4**a,b, violet ALP patches and red BMN patches (stained by Alizarin Red S, ARS) were observed in all groups, suggesting that MC3T3‐E1 cells could express ALP and BMN to some extent. We found that among all the experimental groups, the ALP and BMN expression in the pNBCSMs group was highest (Figure 4d,e), implying that pNBCSMs could promote bone formation. CMCs and Sr in pNBCSMs have a synergistic effect in promoting osteogenesis. Osteocalcin (Ocn) and Osteobridging protein (Opn) are key factors regulating osteoblast metabolism and bone mineralization.^[^ ^37^, ^50^ ^]^ Thus, we evaluated Ocn and Opn expression levels in different groups by immunofluorescence (IF) staining. As shown in Figure 4c, Ocn and Opn in the pNBCSMs group showed the strongest fluorescence intensity, which was further demonstrated by statistical analysis of mean fluorescent intensity (Figure 4f,g). Moreover, ALP, Runx2, and Collgen‐I (Col‐I) are important markers of osteoblast differentiation and bone mineralization.^[^ ^51^, ^52^ ^]^ As shown in Figure 4h,i, the gene expression (ALP, Runx2 and Col‐I) was significantly higher in the pNBCSMs group, indicating the positive effect of pNBCSMs on promoting bone formation. This outcome could be attributed to the osteogenic efficacy of the Sr element in pNBCSMs activating the Wnt/β‐conjugated protein pathway. Furthermore, the expression of oncogene p53 and anti‐apoptotic gene Bcl‐2 showed down‐regulation and up‐regulation, respectively (Figure 4i), which implied that pNBCSMs could inhibit MC3T3‐E1 cell apoptosis, consistent with previous anti‐apoptosis studies. Finally, the expression of the inflammatory gene (MMP‐9) was significantly lower, suggesting that pNBCSMs may have an anti‐inflammatory effect (Figure 4i). In conclusion, these results revealed that pNBCSMs had great potential in bone defect repair.

**Figure 5:**
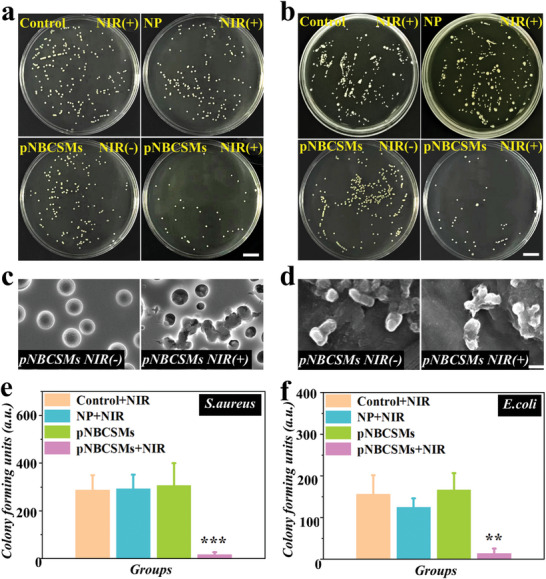
Photothermal antimicrobial effect of pNBCSMs a,b) Images of *S. aureus* (a) and *E. coli* (b) on the agar plate in different groups (*n* = 3). The scale bar represents 14 mm. c,d) SEM images of *S. aureus* (c) and *E. coli* (d) after being treated with pNBCSMs (NIR‐) and pNBCSMs (NIR+), respectively. The scale bar is 0.5 µm. e,f) Statistics of *S. aureus* (e) and *E. coli* (f). **p < 0.01 and ***p < 0.001, one‐way ANOVA using the Tukey post‐hoc test.

Bacterial‐induced infections pose a major challenge during the bone repair process. The photothermal effect has been widely recognized as a safe and efficient anti‐infection strategy.^[^ ^53^ ^]^ We evaluated the photothermal antimicrobial performance of pNBCSMs against *Staphylococcus aureus* (*S. aureus*) and *Escherichia coli* (*E. coli*). Under NIR irradiation, the counts of colonies in the pNBCSMs group (NIR+) were markedly reduced, indicating that the photothermal effect of pNBCSMs could efficiently inactivate *S. aureus* and *E. coli* (**Figure** **5**a,b). The statistical analysis in Figure 5e,f proved this result. In addition, SEM images of the bacteria were also obtained (Figure 5c,d). We observed that the bacterial structure was significantly disrupted after NIR irradiation, whereas the bacteria in the pNBCSMs (NIR‐) group maintained a normal structure.

**Figure 6:**
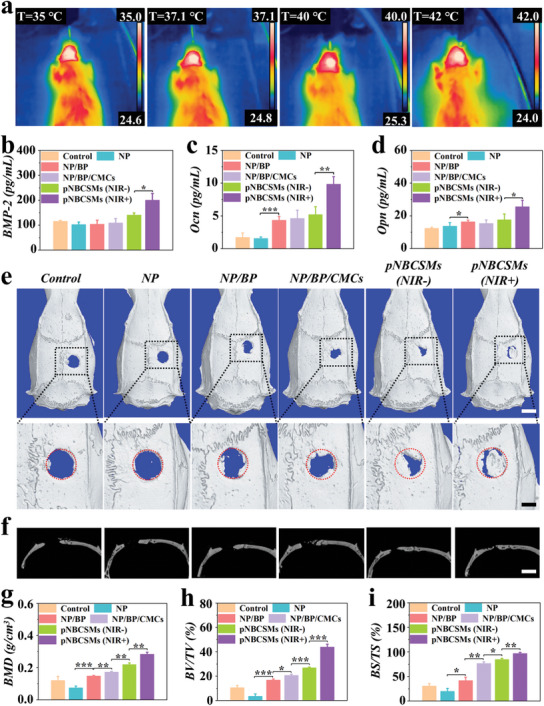
In vivo osteogenesis‐promoting effect of pNBCSMs a) Thermal images of bone defect site implanted with pNBCSMs irradiated with NIR. b–d) The ELISA results of osteogenesis‐related protein in rat serum. e) Representative 3D reconstruction micro‐CT images of skull defect region from rats after different treatments at 8 weeks. The red circle indicates the range of original bone defects. The scale bars are 5 mm (top) and 1.5 mm (bottom). f) Representative 2D micro‐CT images of skull defect region from rats after different treatments at 8 weeks. Scale bar is 3 mm. g–i) Statistical analysis of BMD (g), BV/TV (h) and BS/TS i) of skull defect region from rats after different treatments (*n* = 4). *p < 0.05 and **p < 0.01.

To evaluate the impact of pNBCSMs on bone repair in vivo, we constructed rat cranial bone defect models and injected different materials (NP, NP/BP, NP/BP/CMCs, pNBCSMs) into the bone defect site, wherein rats in the control cohort received a saline solution treatment. When the bone defect site treated with pNBCSMs was exposed to NIR irradiation, the temperature increased from 35 to 42 °C (**Figure** **6**a). At week 8 post‐surgery, we assessed the treatment effect using the ELISA test, micro‐computed tomography CT (micro‐CT), and histological staining. We measured the concentrations of osteogenesis‐related factors in the serum of each group of rats after standardizing the factors with ELISA kits (Figure S10, Supporting Information). As shown in Figure 6b,d, the levels of BMP‐2, Ocn, and Opn increased in pNBCSMs (NIR+) group in comparison with the other groups, suggesting that the treatment of pNBCSMs (NIR+) markedly promoted the production of the osteogenesis‐related factors in rats. Besides, the results of micro‐CT 3D reconstruction and 2D image revealed an obvious reduction in the bone defect area in the pNBCSMs (NIR+) group (Figure 6e,f). Additionally, the bone mineral density (BMD), bone volume/tissue volume (BV/TV) and bone surface/tissue surface (BS/TS) of rats in all groups were analyzed statistically based on micro‐CT results (Figure 6g–i). It was found that the BMD, BV/TV, and BS/TS of the rats in the pNBCSMs (NIR+) group were greater over the other groups, demonstrating the higher new bone quality and faster bone growth rate with pNBCSMs (NIR+) treatment. Interestingly, in contrast to NP/BP group, BMD, BV/TV, and BS/TS were also elevated in the NP/BP/CMCs group. These findings suggested that pNBCSMs irradiated by NIR had a positive bone regeneration capacity, which could be attributed to Sr and CMCs released from pNBCSMs in response to NIR. In addition, the results demonstrated that the BP‐based hydrogel (NP/BP) exhibited a notable osteogenic effect in comparison to hydrogel microspheres without BP (NP). This finding substantiates the hypothesis that BP exerts a pro‐osteoporotic effect on bone repair in vivo. In contrast, the Sr‐loaded hydrogel microspheres (pNBCSMs) demonstrated a more efficacious bone repair‐promoting effect when triggered by near‐infrared light. As mentioned above, the composite hydrogel combines Sr, CMCs and BP, resulting in a synergistic effect that endows the material with multiple bioactivities such as osteogenesis, anti‐inflammatory, anti‐apoptotic and antimicrobial, thus demonstrating an integrated effect.

**Figure 7:**
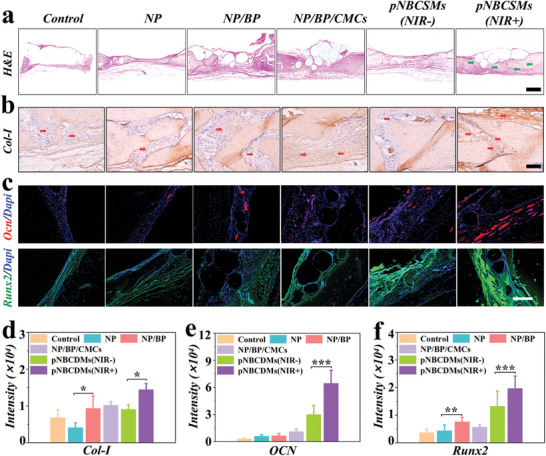
Histological analysis of bone repair a) Representative images of Hematoxylin Eosin staining of skull defect region from rats after different treatments at 8 weeks. The scale bar is 250 µm. b) Representative immunohistochemical staining images of Col‐I of skull defect region from rats after different treatments at 8 weeks. The red arrow shows the Col‐I. The scale bar is 150 µm. c) IF staining images of Ocn and Runx2 of skull defect region from rats after different treatments at 8 weeks. The scale bar is 200 µm. d–f) Statistical analysis of Col‐I (d), OCN (e), and Runx2 (f) (*n* = 3). *p < 0.05, **p < 0.01, and ***p < 0.001.

The osteogenic properties of pNBCSMs were further evaluated by histological staining. As exhibited in **Figure** **7**a, in contrast to the other groups, the pNBCSMs (NIR+) group had conspicuous bone formation (site indicated by the green arrowheads), with smaller bone defects and thicker new bone. In addition, Col‐I, Ocn, and Runx2 were most strongly expressed in the pNBCSMs (NIR+) group, indicating that pNBCSMs irradiated by NIR could enhance bone regeneration through accelerating bone metabolism and osteoblast differentiation. Statistical analysis of these markers further supported these findings (Figure 7d–f). These results indicated that pNBCSMs under NIR irradiation had a promising potential for promoting bone regeneration. Moreover, we explored the pathological pattern in the internal organs of rats in each group by H&E staining to evaluate the biosafety of pNBCSMs in vivo. As shown in the results of Figure S11 (Supporting Information), the organs of rats treated with NP, NP/BP, NP/BP/CMCs, pNBCSMs (NIR‐), and pNBCSMs (NIR+) had no toxic effects, and all the organs demonstrated normal tissues and structures, which suggested a reliable biosafety of pNBCSMs in vivo. As reported in previous studies, Sr is an essential trace element inhibiting bone resorption and increasing bone formation. As is generally known, mineralization is a key step in bone repair: Sr promotes the secretion of bone matrix proteins (e.g., Col‐I) and mineralization‐associated proteins (e.g., Ocn) by osteoblasts, meaning that the newly formed bone matrix can become strong and functional. And CMCs, a polysaccharide‐like organic material, provides a favorable cellular microenvironment for bone regeneration as well as osteogenic effects. So, the synergistic effect of Sr and CMCs together promotes osteogenesis.

**Figure 8:**
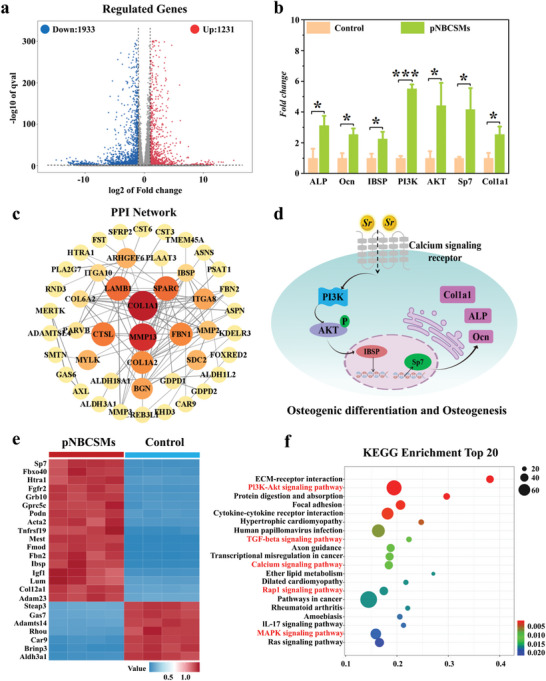
Transcriptomic analysis of osteogenic induction of pNBCSMs a) The volcano plot of the differentially expressed genes identified by transcriptome sequencing, the up‐regulated genes are marked in red color and the down‐regulated genes are marked in blue color. Cutoff: *p*‐value < 0.05 and |log_2_ FC| > 1. b) Real‐time Quantitative PCR (RT‐qPCR) analysis on related gene expression in MC3T3‐E1 cells in pNBCSMs group. *p < 0.05 and ***p < 0.001, one‐way ANOVA using the Tukey post‐hoc test. c) The protein interaction network analysis of the differential genes based on the database STRING. d) Proposed mechanisms of Osteogenesis induced by pNBCSMs to accelerate bone regeneration. e) The heat map of differential genes between the control and pNBCSMs groups, red and blue represent an up‐regulated gene and down‐regulated gene, separately. f) Top 20 signal pathways enriched by differentially expressed genes using KEGG analysis.

To further explore the mechanisms of bone formation, we performed a sequencing analysis of MC3T3‐E1 cells cultured on pNBCSMs. **Figure** **8**a showed that differentially expressed genes (DEGs) in MC3T3‐E1 cells in the pNBCSMs group and blank control group were observed in the volcano plot. Among the 3164 differentially expressed genes identified, there were 1231 up‐regulated genes (log_2_ FC ≥ 1 & q < 0.05) and 1933 down‐regulated genes (log_2_ FC ≤ −1 & q < 0.05). In the differential gene grouping cluster diagram (Figure 8e), the integrin‐binding sialoprotein (IBSP) and Sp7 transcription factor were higher than those of the control group. Figure 8c is a map of the protein‐protein interaction (PPI) network analysis. The results of the PPI enrichment analysis demonstrated a network of proteins associated with MC3T3‐E1 cell proliferation and osteogenic differentiation. Bone matrix protein Col1a1 and IBSP increased significantly. It has been shown that Sp7, IBSP, and Col1a1 are important bone matrix proteins that are closely related to bone formation and development.^[^ ^54^ ^]^ DEGs were further validated to reveal potential possible osteogenic signaling pathways. As shown in Figure 8f, the PI3K‐AKT signaling pathway had the highest enrichment score in the pNBCSMs group, indicating that pNBCSMs could promote bone healing through this signaling pathway. Strontium can promote osteogenesis via the PI3K‐AKT signaling pathway.^[^ ^55^, ^56^ ^]^ The RT‐qPCR result in Figure 8b showed that PI3K and AKT gene expression were significantly higher in the pNBCSMs group, which predicted that pNBDSMs might activate the PI3K‐AKT signaling pathway through the released strontium. In addition, the IBSP and Sp7 genes had higher expression in the pNBCSMs group, attributed to the activated PI3K‐AKT signaling pathway that promotes the expression of the IBSP gene and Sp7 gene. And it contributes to bone matrix proteins Ocn, ALP and Col1a1 synthesis and secretion. This is consistent with previous findings. As reported in previous studies, Sr ions are able to bind to cell‐surface receptors (e.g., integrin receptors) and initiate the activation of PI3K, which further recruits and activates AKT.^[^ ^57^, ^58^ ^]^ Activated AKT promotes the phosphorylation of downstream signaling molecules, such as mTOR, which promotes accelerated proliferation and differentiation of osteoblasts, thereby contributing to new bone formation.^[^ ^59^, ^60^ ^]^ On the other hand, activation of the PI3K‐AKT signaling pathway also promotes the secretion of bone matrix proteins (e.g., Col1a1) and mineralization‐associated proteins (e.g., Ocn) by osteoblasts, thus accelerating the mineralization process of the matrix.^[^ ^61^, ^62^ ^]^ Mineralization is a key step in bone repair, meaning that the newly formed bone matrix can become strong and functional. Finally, AKT protects osteoblasts from apoptosis by inhibiting pro‐apoptotic proteins (e.g., BAD) and activating anti‐apoptotic proteins (e.g., Bcl‐2), thereby increasing cell survival,^[^ ^63^, ^64^ ^]^ which was confirmed in our study. In summary, Sr promotes the proliferation and differentiation of osteoblasts and accelerates matrix mineralization by activating the PI3K‐AKT signaling pathway, thus effectively promoting the process of bone repair. Figure 8d demonstrated the possible mechanism inducing osteogenesis to accelerate bone regeneration by pNBCSMs.

## 3 Conclusion

Overall, the current clinical treatments for bone defects are usually limited by factors such as donor insufficiency, inflammatory response, and integration failure. In response to these challenges, biologically active hydrogel implants have been extensively explored to promote bone tissue regeneration. Although these studies have advanced progress in bone repair, most research on hydrogel implants has focused on organic components such as polysaccharides and proteins, with minimal attention paid to the role of trace elements in bone regeneration. In addition, conventional hydrogel implants largely impede drug penetration to the defected area and form a certain physical obstacle to new tissue growth due to their large volume.

Thus, we presented NIR‐responsive pNBCSMs with monodispersity and porous structures based on microfluidics technology for bone defect repair. By integrating BP with photothermal conversion capability and pNIPAM with thermal responsiveness, the generated pNBCSMs exhibited a stable and repeatable responsiveness to NIR light, contributing to the controllable release of incorporated Sr from pNBCSMs under the NIR trigger. Compared to traditional hydrogel materials, this smart release mechanism provides greater precision and therapeutic control, which enable the adjustment of drug release according to the actual condition of the bone defect area, thus improving the therapeutic effect. In addition, in vitro experiments demonstrated that pNBCSMs had excellent biocompatibility, antimicrobial properties and positive effects on promoting osteoblast differentiation and bone mineralization. Furthermore, animal experiment results revealed that pNBCSMs could accelerate defective bone repair due to the controllable release of Sr under the stimulation of NIR light. Finally, transcriptomic analysis' result revealed that Sr within pNBCSMs promotes the proliferation, differentiation and survival of osteoblasts and accelerates matrix mineralization by activating the PI3K‐AKT signaling pathway, thus effectively promoting the process of bone repair.

In summary, black phosphorus tagged responsive strontium hydrogel particles are innovative for promoting bone regeneration. The developed pNBCSMs demonstrate controllable drug release, superior biocompatibility, powerful bone repair effects and potential personalized treatments, which breaks through the limitations of traditional hydrogel biomaterials and positioning themselves as a valuable addition to the clinical applications.

## 4 Experimental Section

### Materials

Poly(N‐isopropylacrylamide) (pNIPAM), N‐Methylenebisacrylamide (Bis), and HMPP were obtained from the Sigma‐Aldrich supplier. The carboxymethyl chitosan (CMCs) was bought from a chemical supplier in Shanghai, China called Shanghai Macklin Biochemical Technology Co. The anhydrous form of strontium chloride (SrCl_2_) was sourced from Shanghai Aladdin Biochemical Technology Co. in China. Black phosphorus (BP) was purchased from XFNANO Materials Tech CO., Ltd (Nanjing, China). α‐MEM cell culture medium was bought from Hyclone Corporation, USA. Cell Counting Kit‐8 (CCK8), Calcein/PI Cell Viability/Cytotoxicity Assay Kit, cDNA Synthesis Kit and q‐PCR low ROX were all bought from Beyotime Institute of Biotechnology (Shanghai, China). Osteocalcin (BGLAP) (Ocn) antibody and osteopontin (Opn) antibody were purchased from ABclonal Biotechnology Ltd. (Wuhan, China). Sprague Dawley (SD) rats that were 10 weeks old in this study were acquired from AUNOCO Biotechnology Co. (Nanjing, China). Deionized water (>18.2 MΩ·cm) was used in this study. All chemicals were analytical grade and used as specified.

### Microfluidic Device Fabrication

The microfluidic device consisted of two glass capillaries and a syringe needle, which were assembled on a slide. The inner and outer capillaries had diameters of 750 and 580 µm, respectively. To create a smooth opening, a micropipette puller (Sutter Instrument Co., Novato) was utilized to thin the middle of the capillary gradually until it broke and then sanded the openings with fine sandpaper. The connections of the microfluidic device were bonded with epoxy adhesive.

### Preparation of pNBCSMs

The pNBCSMs precursor solution was prepared by mixing 10% (w/v) pNIPAM, 0.34% (w/v) BIS, 0.5% (w/v) CMCs, 5% (w/v) SrCl_2_, 0.075 mg mL^−1^ BP and 5% (v/v) HMPP. Under the control of a syringe pump (PHD 2000, Harvard Apparatus), the hydrogel precursor solution (inner phase) and the oil containing liquid paraffin and span (9:1 v/v) (outer phase) were at rates of 0.8 and 2 mL h^−1^, respectively. Within a microfluidic device, the hydrogel precursor was split into individual droplets by the shear force of the outer phase, followed by photopolymerization under UV. The obtained hydrogel microspheres were washed repeatedly with deionized water and freeze‐dried for subsequent use.

### Characterization

The attenuated total reflection (ATR) patterns of pNIPAM hydrogel (NP), pNIPAM hydrogel only with Sr (NP/Sr), pNIPAM hydrogel with BP and CMCs (NP/BP/CMCs), pNIPAM hydrogel with BP, CMCs and Sr (NP/BP/CMCs@Sr) were tested by Fourier Transform Infrared Spectroscopy (FTIR Spectroscopy). The hydrogels' microstructure was detected by SEM (Zeiss, Merlin). Elemental composition and distribution on the surface of pNBCSMs were detected by Energy Dispersive Spectroscopy (EDS). The hydrophilicity of the samples was assessed through contact angle measurements using specialized equipment (VCA OPTIMA, AST). The rheological properties of the hydrogels were examined using a rotational rheometer (ThermoFisher Scientific, HAAKE MARS 40). A material mechanical testing system (MTS Standard 43) was utilized to assess the mechanical properties of the samples, during which the stress‐strain curve was recorded (compression rate: 5 mm min^−1^). This test was repeated 3 times. Based on previous research methods, the hydrogels' swelling ratio was detected according to the calculation Equation (1):

(1) Swellingrate=(St−S0)/S0×100%

where S_0_ and S_t_ represented the initial weight of the lyophilized hydrogel and the water‐absorbed swollen hydrogel, respectively.

The hydrogels' degradation behavior was measured based on the calculation Equation (2):

(2) Remainingweightratio=Wt/W0×100%

where W_0_ represented the initial weight of the lyophilized hydrogel and W_t_ referred to the remaining weight of the lyophilized hydrogels after degradation, respectively.

### NIR Responsiveness of pNBCSMs

The pNBCSMs containing different concentrations of BP were vertically irradiated with NIR (808 nm) at different powers, and its' temperature was measured by an infrared camera (FLIR Systema, E5xt). Besides, to determine the volume variation of pNBCSMs triggered by NIR, the pNBCSMs were irradiated with NIR (808 nm, 3.45 W cm^−2^) for 1 min before being brought back to their initial condition at ambient temperature, followed by the next irradiation. The aforementioned procedure was conducted for 10 cycles, and the morphology of pNBCSMs was recorded by optical microscopy. The diameters of the pNBCSMs were measured before and after NIR irradiation using Image J software.

### In Vitro Drug Release Experiment

1 mg/mL phycoerythrin (PE) was mixed with prepared pNBCSMs. To explore the drug release properties of pNBCSMs triggered by NIR, namely the pNBCSMs (NIR+) group exposed to NIR and the pNBCSMs (NIR‐) group without NIR treatment were performed. Specifically, the samples in the pNBCSMs (NIR+) group were exposed to NIR light for a duration of 5 min, followed by a recovery period at room temperature before subsequent NIR irradiation, while the samples in the pNBCSMs (NIR‐) group received no treatment. Images of fluorescence were captured following every exposure round, and the total amount of polyethylene release was accurately measured using Image J program for analysis. As for Sr^2+^ release test, pNBCSMs prepared from 1 mL of pNBCSMs precursor solution were placed in 5 mL of PBS (pH 7.4) in a 37 °C environment. Samples in the pNBCSMs (NIR+) group were irradiated under NIR for 5 min and then recovered at room temperature for a period of time, while samples in the pNBCSMs (NIR‐) group were left untreated. The above operation was repeated every other day. Hundred microliters was removed from the above solution and then the concentration of Sr in the solution was examined by Inductively Coupled Plasma Mass Spectrometry (ICP‐MS) (Agilent 7700X) to determine the rate of Sr ion release, and finally 100 uL of PBS was added to the above solution to maintain a constant volume.

### Cell Proliferation and Apoptosis Detection

α‐MEM culture medium was used for MC3T3 cells. After being inoculated for 24 h, cells were intervened with pNBCSMs' extracts containing CMCs (0.2% CMCs, 0.5% CMCs, 0.8% CMCs) and NP hydrogel microspheres extracts, respectively. Then, cell viability was detected on days 0, 1, and 3 using the CCK8 kit. Additionally, MC3T3‐E1 cells were grouped into five (control group, NP group, NP/BP/CMCs, pNBCSMs group, and positive group (induction of apoptosis in MC3TC‐E1 cells with hydrogen peroxide)) with different treatments. After incubation for 5 days, MC3T3‐E1 cells were tested with an apoptosis detection kit, and ≈2 × 10^4^ cells were analyzed using flow cytometry (FACSCanto, BD, USA).

### Real‐Time Quantitative PCR (RT‐qPCR)

MC3T3‐E1 cells' RNA was extracted by applying RNAiso plus according to the instructions. The concentration of total RNA extracted from samples was determined in each group using Nanodro 2000 and synthesized cDNA. The synthesized cDNAs were amplified using an RT‐qPCR system (Applied Biosystems). Expression levels of genes (Collagen‐I (Col‐I), Runx2, ALP, MMP‐9, P53, Bcl‐2) were normalized using GAPDH. Primers are shown in Table S1 (Supporting Information).

### Osteogenic Induction and Detection

MC3T3‐E1 cells were treated with hydrogel microsphere extracts, after inoculated for 24 h. Following being cultured with medium containing osteogenic differentiation induction solution for 14 days, the cells' potential for osteogenic differentiation was tested utilizing ALP Staining Kit. The resultant stains were documented using a Nikon camera, and were analyzed using Image J software. Bone mineralization staining was used to detect the mineralization ability of MC3T3‐E1 cells. After being cultured for 21 days, the bone mineralization capacity of MC3T3‐E1 cells was assayed using the ARS Staining Kit, which was photographed with a camera (Nikon, Japan) and analyzed using Image J software.

### Immunofluorescence Staining

Put it simply, MC3T3‐E1 cells were incubated separately with Ocn Rabbit Polyclonal Antibody (1:100 dilutions) and Opn Rabbit Antibody (1:50 dilutions) overnight at 4 °C. Subsequently, they were exposed to FITC‐Labeled secondary antibody individually for 60 min. Finally, the nucleus was stained with DAPI staining solution and treated with anti‐fluorescence quenching. Pictures were taken with a LEICA fluorescence microscope.

### Antibacterial Property Test

Antibacterial activity of pNBCSMs was evaluated using *S. aureus* and *E. coli*. In brief, the 48‐well plates were filled with NP hydrogel microspheres and pNBCSMs. Subsequently, a droplet of 10 microliters containing a bacterial suspension of 1 × 10^8^ colony‐forming units per milliliter was introduced, wherein the wells without hydrogel were set as blank control groups. Three groups were exposed to NIR (808 nm, 3.45 W cm^−2^) for 5 min, then 1 mL sterile PBS was added to each well to resuspend bacterial solution. Finally, 20 µL bacterial solution was taken to coat the plate and the count of colonies was recorded following an 18 h incubation at 37 °C. The experiment was repeated 5 times. Morphology of *S. aureus* and *E. coli* after different treatments was captured by SEM.

### Animal Model Establishment

Animal experiments were authorized by the Animal Experimentation Ethics Committee of Nanjing University Medical School (2023AE01019). Rats participating in the research were assigned at random to the control group, NP hydrogel microspheres (shortened to NP) group, NP/BP hydrogel microspheres (NP/BP) group, NP/BP/CMCs hydrogel microspheres (NP/BP/CMCs) group, pNBCSMs (NIR‐) group, and pNBCSMs (NIR+) group (*n* = 4). Then, a 3 mm circular full‐length bone defect was created at the center of the right parietal bone by a circular hollow bone drill. After that, sterilized materials were injected into the site of bone defects in each corresponding experimental group, notably, rats in the control cohort received a saline solution treatment. Of note, penicillin was administered intramuscularly to rats for 3 days after surgery to prevent infection. After 8 weeks of treatment, the mice in the research lab were compassionately euthanized, and the defects were exposed along the original incision to observe the repair of the bone defects. Rat skull samples were fixed in 4% PFA for later micro‐CT test. Subsequently, the specimens underwent decalcification using a 12% solution of Ethylene Diamine Tetraacetate (EDTA) and were subsequently dehydrated in ethanol for histological staining, including H&E staining, immunofluorescence staining for Ocn and Runx2, and immunohistochemical staining for Col‐I.

### Transcriptome Sequencing and Analysis

MC3T3‐E1 was cultured for 48 h. Then, the medium was replaced with osteogenic induction medium and pNBCSMs extract. Following a week of differentiation, the cells were treated with Trizol reagent to break them down and the lysate products were sequenced for transcriptomics. Transcriptome sequencing and analysis were performed by LC‐Bio Technologies (Hangzhou) Co.

### Statistical Analysis

In this research study, the data was reported using the mean ± SD format detailed in the figure legends, with statistical significance determined through Student's t‐test and one‐way ANOVA analyses. Statistical significance was determined for results with a *p*‐value lower than 0.05. Statistical Product and Service Solutions 20.0 (SPSS 20.0) software was used to statistically analyze.

## Conflict of Interest

The authors declare no conflict of interest.

## Author Contributions

Y.J.Z. and Y.X.W. conceived the conceptualization and designed the experiment; Z.W.L. carried out the experiments; and Z.W.L., H.Z., and J.J.G. wrote the paper.

## Supporting information



Supporting Information

Supplemental Table 1

## Data Availability

The data that support the findings of this study are available from the corresponding author upon reasonable request.

## References

[1] X. Han , Q. Saiding , X. Cai , Y. Xiao , P. Wang , Z. Cai , X. Gong , W. Gong , X. Zhang , W. Cui , Nanomicro Lett. 2023, 15, 239. 37907770 10.1007/s40820-023-01187-2 PMC10618155

[2] M. Favero , E. Belluzzi , A. Ortolan , M. Lorenzin , F. Oliviero , A. Doria , C. R. Scanzello , R. Ramonda , Nat. Rev. Rheumatol. 2022, 18, 171. 35105980 10.1038/s41584-021-00747-3

[3] Q. Yao , X. Wu , C. Tao , W. Gong , M. Chen , M. Qu , Y. Zhong , T. He , S. Chen , G. Xiao , Signal Transduction Targeted Ther. 2023, 8, 56. 10.1038/s41392-023-01330-w PMC9898571 36737426

[4] L. Wu , L. Cheng , J. Yang , Y. Yan , E. Zhang , Z. Kochovski , L. Li , Z. Wang , L. Deng , Y. Lu , P. Besenius , W. Cui , G. Chen , Adv. Mater. 2022, 34, 2207526. 10.1002/adma.202207526 36103707

[5] Z. Zhao , G. Li , H. Ruan , K. Chen , Z. Cai , G. Lu , R. Li , L. Deng , M. Cai , W. Cui , ACS Nano. 2021, 15, 13041. 34342981 10.1021/acsnano.1c02147

[6] B. Zhang , J. D. Skelly , J. R. Maalouf , D. C. Ayers , J. Song , Sci. Transl. Med. 2019, 11, aau7411. 10.1126/scitranslmed.aau7411 31341064

[7] A. E. Jakus , A. L. Rutz , S. W. Jordan , A. Kannan , S. M. Mitchell , C. Yun , K. D. Koube , S. C. Yoo , H. E. Whiteley , C. P. Richter , R. D. Galiano , W. K. Hsu , S. R. Stock , E. L. Hsu , R. N. Shah , Sci. Transl. Med. 2016, 8, 358ra127. 10.1126/scitranslmed.aaf7704 27683552

[8] Z. Zhao , G. Li , H. Ruan , K. Chen , Z. Cai , G. Lu , R. Li , L. Deng , M. Cai , W. Cui , ACS Nano. 2021, 15, 13041. 34342981 10.1021/acsnano.1c02147

[9] V. Iotsova , J. Caamaño , J. Loy , Y. Yang , A. Lewin , R. Bravo , Nat. Med. 1997, 3, 1285. 9359707 10.1038/nm1197-1285

[10] H. Zhang , D. Xu , Y. Zhang , M. Li , R. Chai , Smart Med. 2022, 1, 20220011. 10.1002/SMMD.20220011 PMC11235963 39188746

[11] Z. K. Cui , S. Kim , J. J. Baljon , B. M. Wu , T. Aghaloo , M. Lee , Nat. Commun. 2019, 10, 3523. 31388014 10.1038/s41467-019-11511-3 PMC6684526

[12] J. Yang , Z. Chen , C. Gao , J. Liu , K. Liu , X. Wang , X. Pan , G. Wang , H. Sang , H. Pan , W. Liu , C. Ruan , Nat. Commun. 2024, 15, 3565. 38670999 10.1038/s41467-024-48023-8 PMC11053166

[13] H. Zhou , K. Yu , H. Jiang , R. Deng , L. Chu , Y. Cao , Y. Zheng , W. Lu , Z. Deng , B. Liang , Biomacromolecules. 2021, 22, 4552. 34590825 10.1021/acs.biomac.1c00842

[14] Z. Liu , W. Tang , J. Liu , Y. Han , Q. Yan , Y. Dong , X. Liu , D. Yang , G. Ma , H. Cao , Bioact. Mater. 2023, 20, 610. 35846848 10.1016/j.bioactmat.2022.06.008 PMC9256661

[15] A. S. Mao , J. Shin , S. Utech , H. Wang , O. Uzun , W. Li , M. Cooper , Y. Hu , L. Zhang , D. A. Weitz , D. J. Mooney , Nat. Mater. 2017, 16, 236. 27798621 10.1038/nmat4781 PMC5372217

[16] X. Zhao , S. Liu , L. Yildirimer , H. Zhao , R. Ding , H. Wang , W. Cui , D. Weitz , Adv. Funct. Mater. 2016, 26, 2809.

[17] X. Chen , L. Ren , H. Zhang , Y. Hu , M. Liao , Y. Shen , K. Wang , J. Cai , H. Cheng , J. Guo , Y. Qi , H. Wei , X. Li , L. Shang , J. Xiao , J. Sun , R. Chai , Smart Med. 2023, 2, 20220038. 10.1002/SMMD.20220038 PMC11235853 39188281

[18] L. Fu , L. Li , Q. Bian , B. Xue , J. Jin , J. Li , Y. Cao , Q. Jiang , H. Li , Nature. 2023, 618, 740. 37344650 10.1038/s41586-023-06037-0

[19] M. Wang , S. Sun , G. Dong , F. Long , J. T. Butcher , Proc Natl. Acad. Sci. USA. 2023, 120, 2081937176. 10.1073/pnas.2213030120 PMC9974439 36791112

[20] X. Zhao , Y. Liu , C. Shao , M. Nie , Q. Huang , J. Li , L. Sun , Y. Zhao , Adv. Sci. 2019, 6, 1901280. 10.1002/advs.201901280 PMC6794614 31637165

[21] S. Lyu , Z. Dong , X. Xu , H. P. Bei , H. Y. Yuen , C. C. James , M. S. Wong , Y. He , X. Zhao , Bioact. Mater. 2023, 27, 303. 37122902 10.1016/j.bioactmat.2023.04.003 PMC10140753

[22] M. Yu , L. Xu , F. Tian , Q. Su , N. Zheng , Y. Yang , J. Wang , A. Wang , C. Zhu , S. Guo , X. Zhang , Y. Gan , X. Shi , H. Gao , Nat. Commun. 2018, 9, 2607. 29973592 10.1038/s41467-018-05061-3 PMC6031689

[23] B. Zhao , X. Li , H. Xu , Y. Jiang , D. Wang , R. Liu , Int J Nanomedicine. 2020, 15, 1797. 32214812 10.2147/IJN.S244815 PMC7083628

[24] B. Yuan , L. Wang , R. Zhao , X. Yang , X. Yang , X. Zhu , L. Liu , K. Zhang , Y. Song , X. Zhang , Sci. Adv. 2020, 6, abc4704. 10.1126/sciadv.abc4704 PMC7732183 33310848

[25] X. Wang , Y. Yu , C. Yang , C. Shao , K. Shi , L. Shang , F. Ye , Y. Zhao , Adv. Funct. Mater. 2021, 31, 2105190.

[26] J. Long , Z. Yao , W. Zhang , B. Liu , K. Chen , L. Li , B. Teng , Du X. F. , C. Li , X. F. Yu , L. Qin , Y. Lai , Adv. Sci. (Weinh). 2023, 10, 2302539. 37616380 10.1002/advs.202302539 PMC10558667

[27] M. Wu , H. Liu , D. Li , Y. Zhu , P. Wu , Z. Chen , F. Chen , Y. Chen , Z. Deng , L. Cai , Adv. Sci. (Weinh). 2024, 11, 2304641. 37933988 10.1002/advs.202304641 PMC10787108

[28] Y. Miao , X. Liu , J. Luo , Q. Yang , Y. Chen , Y. Wang , Adv. Sci. (Weinh). 2024, 11, 2303637. 37949678 10.1002/advs.202303637 PMC10767401

[29] Z. Fei , N. Gupta , M. Li , P. Xiao , X. Hu , Sci. Adv. 2023, 9, adg9933. 10.1126/sciadv.adg9933 PMC10171811 37163589

[30] X. Lin , L. Cai , X. Cao , Y. Zhao , Smart Med. 2023, 2, 20220019. 10.1002/SMMD.20220019 PMC11235688 39188280

[31] D. Liang , G. Kuang , X. Chen , J. Lu , L. Shang , W. Sun , Smart Med. 2023, 2, 20230016. 10.1002/SMMD.20230016 PMC11236066 39188343

[32] H. Geng , Q. Xu , M. Wu , H. Ma , P. Zhang , T. Gao , L. Qu , T. Ma , C. Li , Nat. Commun. 2019, 10, 1512. 30944322 10.1038/s41467-019-09535-w PMC6447597

[33] M. H. Choi , A. Blanco , S. Stealey , X. Duan , N. Case , S. A. Sell , M. F. Rai , S. P. Zustiak , Polymers (Basel). 2020, 12, 1712. 32751604 10.3390/polym12081712 PMC7464943

[34] Y. Chen , Y. Zhou , Z. Hu , W. Lu , Z. Li , N. Gao , N. Liu , Y. Li , J. He , Q. Gao , Z. Xie , J. Li , Y. He , Nanomicro Lett. 2023, 16, 34. 38019305 10.1007/s40820-023-01225-z PMC10686972

[35] C. Yang , Y. Yu , L. Shang , Y. Zhao , Nat. Chem. Eng. 2024, 1, 87.

[36] X. Zhao , Y. Liu , C. Shao , M. Nie , Q. Huang , J. Li , L. Sun , Y. Zhao , Adv. Sci. 2019, 6, 1901280. 10.1002/advs.201901280 PMC6794614 31637165

[37] L. Yang , Y. Liu , L. Sun , C. Zhao , G. Chen , Y. Zhao , Nanomicro Lett. 2021, 14, 4. 34859316 10.1007/s40820-021-00747-8 PMC8639896

[38] H. Chen , F. Bian , L. Sun , D. Zhang , L. Shang , Y. Zhao , Adv. Mater. 2020, 32, 2005394. 10.1002/adma.202005394 33184956

[39] W. Zhou , X. Ma , J. Xiao , X. He , C. Liu , X. Xu , T. Viitala , J. Feng , H. Zhang , Small Sci. 2024, 4, 2300192. 10.1002/smsc.202300192 PMC11935110 40212695

[40] J. Shen , C. Liu , P. Yan , M. Wang , L. Guo , S. Liu , J. Chen , J. M. Rosenholm , H. Huang , R. Wang , H. Zhang , Research (Wash D C). 2022, 2022, 9794235. 35958106 10.34133/2022/9794235 PMC9343082

[41] C. Yang , Y. Yu , L. Shang , Y. Zhao , Nat. Chem. Eng. 2024, 1, 87.

[42] X. Wu , H. Zhu , C. Song , Q. Tan , Y. Zhao , L. Shang , Adv. Mater. 2024, 36, 2309719. 10.1002/adma.202309719 37985138

[43] Z. Zheng , L. Hu , Y. Ge , J. Qi , Q. Sun , Z. Li , L. Lin , B. Tang , Adv. Healthcare Mater. 2022, 11, 2200398. 10.1002/adhm.202200398 35481900

[44] Z. Liu , X. Mo , F. Ma , S. Li , G. Wu , B. Tang , L. Lin , Carbohydr. Polym. 2021, 261, 117869. 33766356 10.1016/j.carbpol.2021.117869

[45] T. Su , M. Zhang , Q. Zeng , W. Pan , Y. Huang , Y. Qian , W. Dong , X. Qi , J. Shen , Bioact. Mater. 2021, 6, 579. 33005823 10.1016/j.bioactmat.2020.09.004 PMC7509181

[46] J. Guo , Z. Luo , F. Wang , H. Gu , M. Li , Smart Med. 2022, 1, 20220003. 10.1002/SMMD.20220003 PMC11235791 39188750

[47] M. Ran , Y. Deng , J. Yan , A. Zhang , Y. Wei , X. Li , H. He , J. Gou , T. Yin , X. Tang , J. Kong , H. Zhang , H. Zhang , Y. Zhang , Chem. Eng. J. 2022, 450, 138291.

[48] X. Yao , G. Zhu , P. Zhu , J. Ma , W. Chen , Z. Liu , T. Kong , Adv. Funct. Mater. 2020, 30, 1909389.

[49] R. Yang , G. Li , C. Zhuang , P. Yu , T. Ye , Y. Zhang , P. Shang , J. Huang , M. Cai , L. Wang , W. Cui , L. Deng , Sci. Adv. 2021, 7, abg3816. 10.1126/sciadv.abg3816 PMC8221628 34162547

[50] S. Chen , X. Han , Y. Cao , W. Yi , Y. Zhu , X. Ding , K. Li , J. Shen , W. Cui , D. Bai , Adv. Funct. Mater. 2024, 34, 2308205.

[51] C. Sun , W. Lan , B. Li , R. Zuo , H. Xing , M. Liu , J. Li , Y. Yao , J. Wu , Y. Tang , H. Liu , Y. Zhou , Stem Cell Res. Ther. 2019, 10, 357. 31779679 10.1186/s13287-019-1440-5 PMC6883626

[52] X. Zhang , W. Jiang , C. Xie , X. Wu , Q. Ren , F. Wang , X. Shen , Y. Hong , H. Wu , Y. Liao , Y. Zhang , R. Liang , W. Sun , Y. Gu , T. Zhang , Y. Chen , W. Wei , S. Zhang , W. Zou , H. Ouyang , Nat. Commun. 2022, 13, 5211. 36064711 10.1038/s41467-022-32868-y PMC9445030

[53] C. Song , X. Wu , Y. Wang , J. Wang , Y. Zhao , Small. 2023, 20, 2310444. 10.1002/smll.202310444 38050927

[54] J. Long , Z. Yao , W. Zhang , B. Liu , K. Chen , L. Li , B. Teng , Du X. F. , C. Li , X. F. Yu , L. Qin , Y. Lai , Adv. Sci. (Weinh). 2023, 10, 2302539. 37616380 10.1002/advs.202302539 PMC10558667

[55] S. Xiao , J. Wei , S. Jin , X. Xia , L. Yuan , Q. Zou , Y. Zuo , J. Li , Y. Li , Adv. Funct. Mater. 2023, 33, 2208968.

[56] S. Li , Y. Sun , Y. Chen , J. Lu , G. Jiang , K. Yu , Y. Wu , Y. Mao , H. Jin , J. Luo , S. Dong , B. Hu , Y. Ding , A. Liu , Y. Shen , G. Feng , S. Yan , Y. He , R. Yan , ACS Appl. Mater. Interfaces. 2023, 15, 4652. 36698266 10.1021/acsami.2c16584

[57] S. Xiao , J. Wei , S. Jin , X. Xia , L. Yuan , Q. Zou , Y. Zuo , J. Li , Y. Li , Adv. Funct. Mater. 2023, 33, 2208968.

[58] S. Li , Y. Sun , Y. Chen , J. Lu , G. Jiang , K. Yu , Y. Wu , Y. Mao , H. Jin , J. Luo , S. Dong , B. Hu , Y. Ding , A. Liu , Y. Shen , G. Feng , S. Yan , Y. He , R. Yan , ACS Appl. Mater. Interfaces. 2023, 15, 4652. 36698266 10.1021/acsami.2c16584

[59] Q. Wu , L. Hu , R. Yan , J. Shi , H. Gu , Y. Deng , R. Jiang , J. Wen , X. Jiang , Bone Res. Res. 2022, 10, 55. 10.1038/s41413-022-00224-x PMC9399250 35999199

[60] L. Qiao , X. Liu , Y. He , J. Zhang , H. Huang , W. Bian , M. M. Chilufya , Y. Zhao , J. Han , Int. J. Mol. Sci. 2021, 22, 11932. 34769367 10.3390/ijms222111932 PMC8584317

[61] H. Yu , P. Wang , H. Lu , J. Guan , F. Yao , T. Zhang , Q. Wang , Z. Wang , BMC Oral Health. 2023, 23, 422. 37365568 10.1186/s12903-023-03040-9 PMC10294445

[62] D. S. Amarasekara , S. Kim , J. Rho , Int. J. Mol. Sci. 2021, 22, 2851. 33799644 10.3390/ijms22062851 PMC7998677

[63] Y. Mao , J. Wang , F. Yu , J. Cheng , H. Li , C. Guo , X. Fan , Drug Des. Dev. Ther. 2015, 9, 5385. 10.2147/DDDT.S89096 PMC4592035 26451091

[64] X. Liang , Y. Ding , Y. Zhang , Y. H. Chai , J. He , S. M. Chiu , F. Gao , H. F. Tse , Q. Lian , Cell Death Dis. 2015, 6, 1765. 10.1038/cddis.2015.91 PMC4669719 25996292

