# Supplementary material for: Black Phosphorus Tagged Responsive Strontium Hydrogel Particles for Bone Defect Repair
Source: Adv Sci (Weinh). 2024 Nov 6;12(1):2408284. doi: 10.1002/advs.202408284 (PMC11714197; doi:10.1002/advs.202408284)
Supplement: Supplementary file 1 — Supporting Information [file ADVS-12-2408284-s002.docx]

**Black phosphorus tagged responsive** **strontium hydrogel particles for bone defect repair**

Zhengwei Liu^1^, Hui Zhang^2^, Jingjing Gan^2,^*, Yuanjin Zhao^2,3,^*, Yongxiang Wang^1,4,^*

^1^ Department of Orthopedics, Northern Jiangsu People's Hospital, Clinical Teaching Hospital of Medical School, Nanjing University, Yangzhou, 225001, China

^2^ Department of Rheumatology and Immunology, Nanjing Drum Tower Hospital, School of Biological Science and Medical Engineering, Southeast University, 210096, Nanjing, China

^3^ Shenzhen Research Institute, Southeast University, Shenzhen 518071, China

^4^ Department of Orthopedics, Northern Jiangsu People's Hospital, Yangzhou, 225001, China

Email: ganjingjing@njglyy.com (J. J. Gan); [yjzhao@seu.edu.cn](mailto:yjzhao@seu.edu.cn) (Y. J. Zhao); [wangyongxiang@nju.edu.cn](mailto:wangyongxiang@nju.edu.cn) (Y.X. Wang)

***
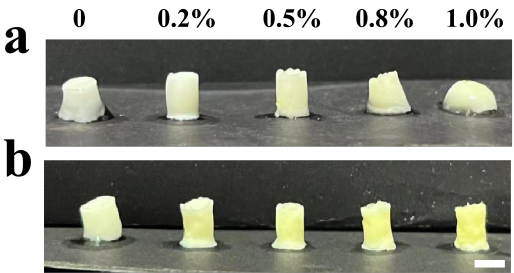
***

**Fig. S1 Preparation and characterization of the pNBCSMs** Photographs of NP/BP/CMCs hydrogel **(a)** and NP/BP/CMCs@Sr hydrogel **(b)** integrating different concentrations of CMCs (0%, 0.2%, 0.5%, 0.8%, and 1.0%). The scale bar represents 5 mm.

***
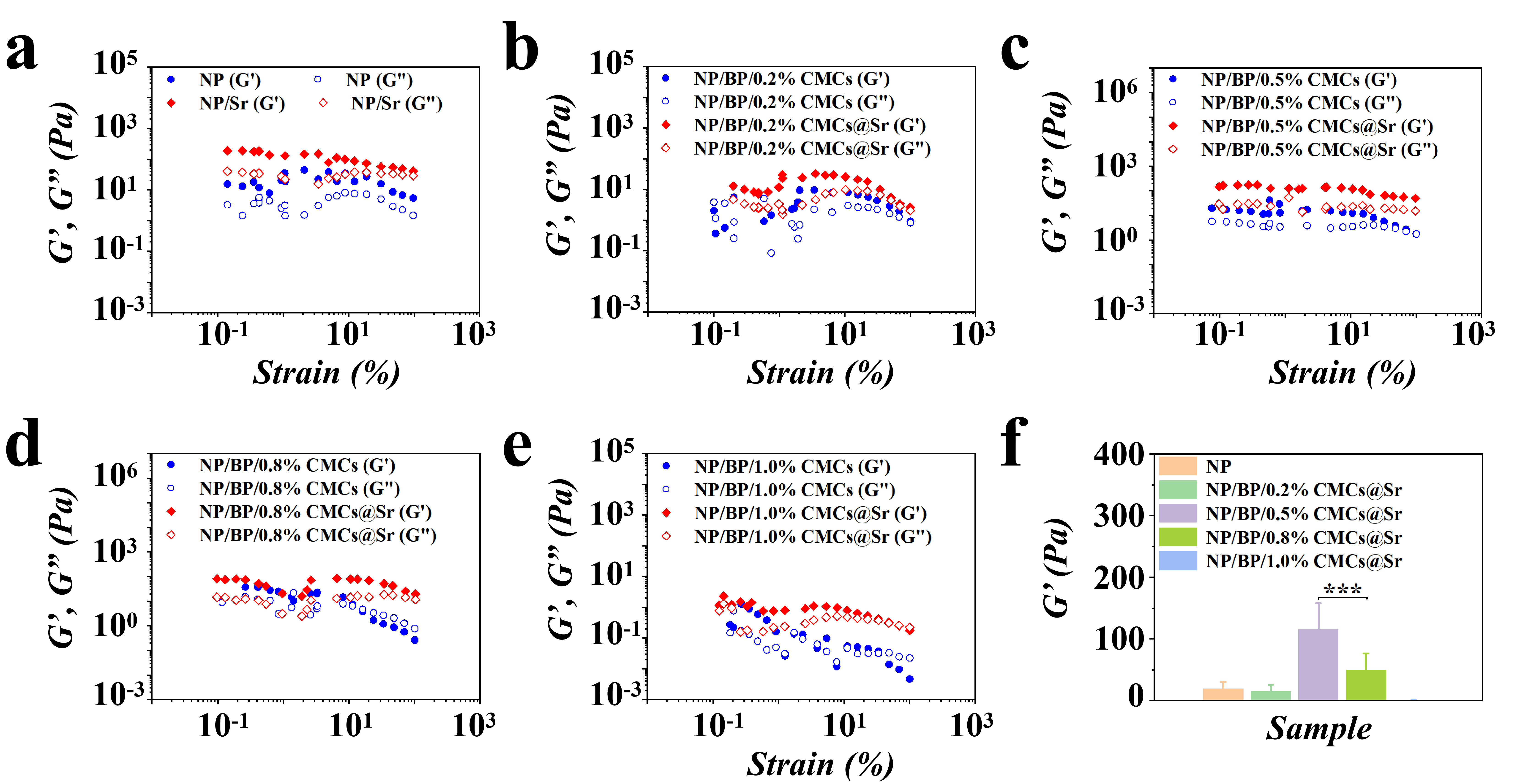
***

**Fig. S2 Preparation and characterization of the pNBCSMs (a-f)** Rheological behaviors of the gels under a strain of 0.1-100%. ****p* < 0.001.

***
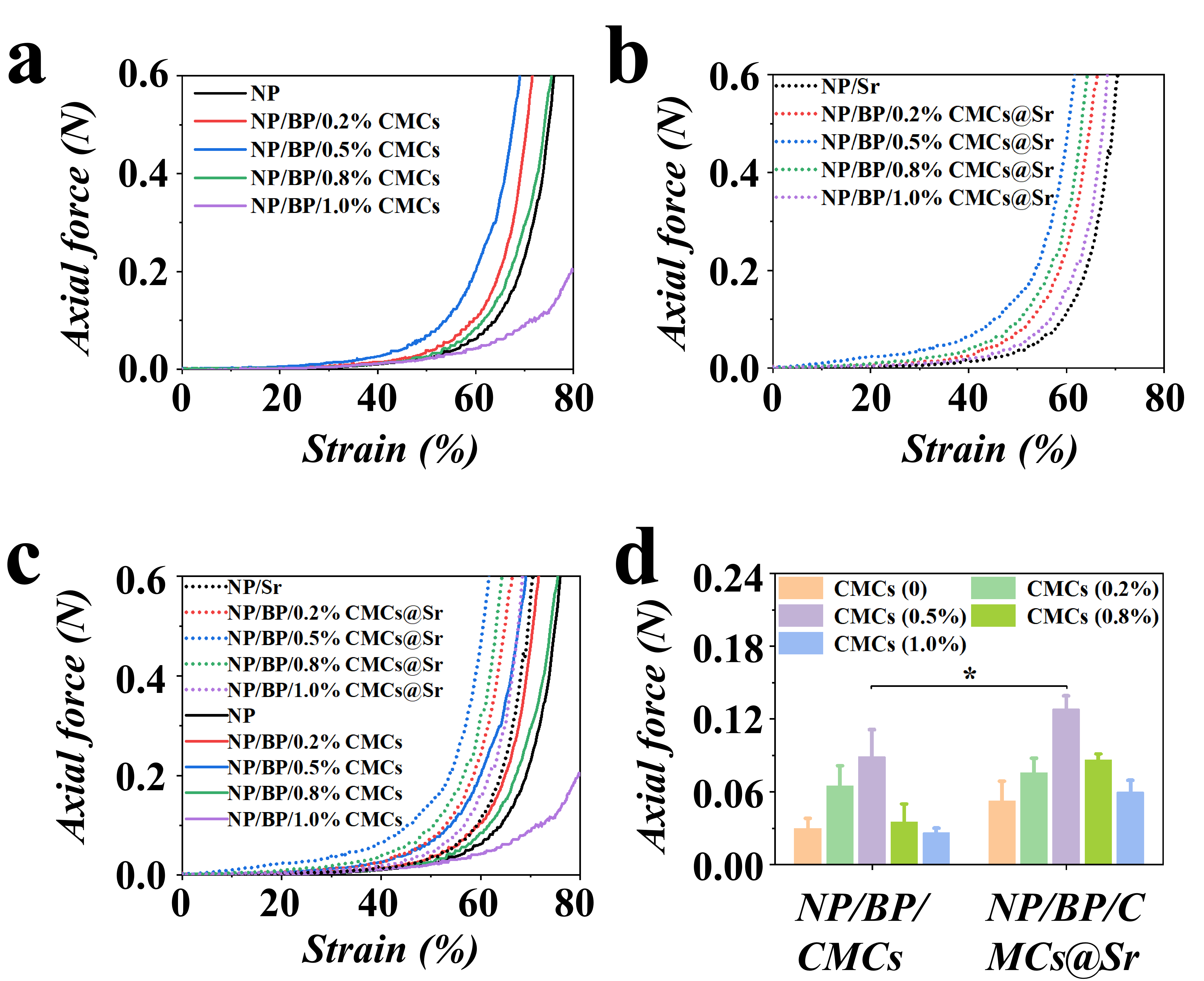
***

**Fig. S3 Preparation and characterization of the pNBCSMs (a-c)** Typical force-strain profiles of the hydrogels. **(d)** Statistic analysis of the axial force of the hydrogels under a strain of 70%.


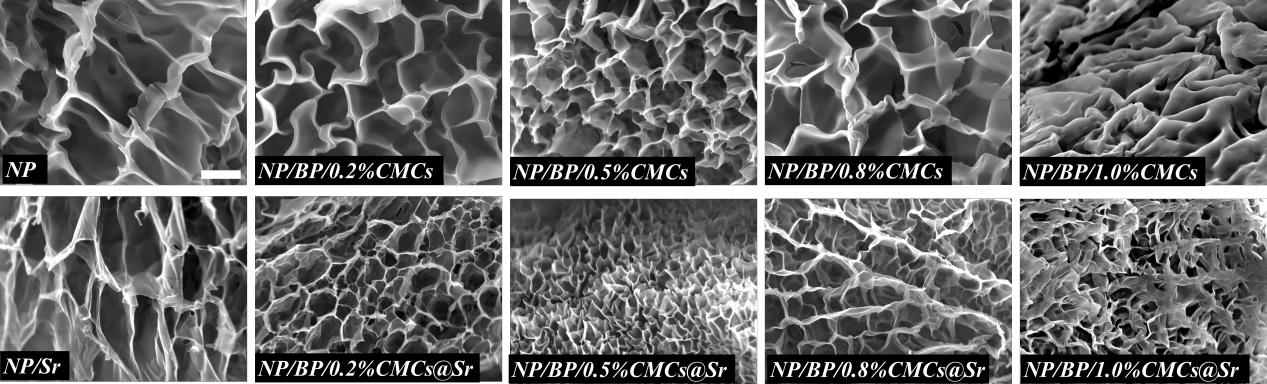


**Fig. S4** **Preparation and characterization of the pNBCSMs** SEM images of the prepared hydrogels. The scale bar represents 40 µm.


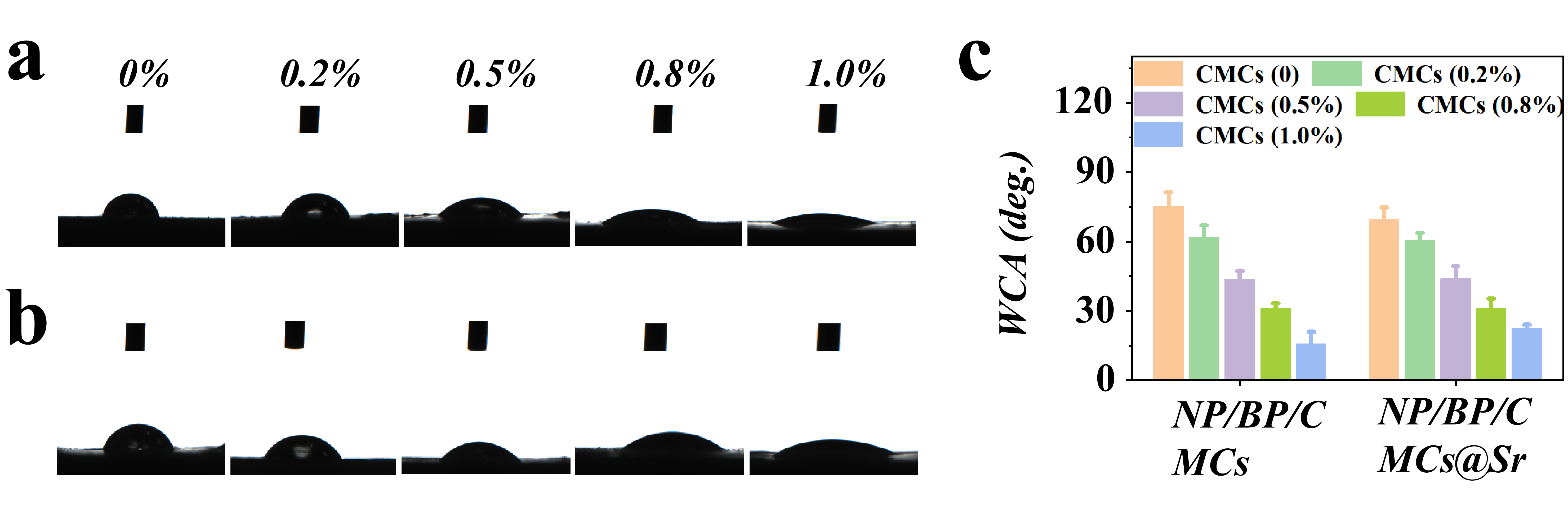


**Fig. S5 Preparation and characterization of the pNBCSMs** Water contact angle (WCA) of NP/BP/CMCs hydrogel **(a)** and NP/BP/CMCs@Sr hydrogel **(b)** integrating different concentrations of CMCs (0%, 0.2%, 0.5%, 0.8%, and 1.0%), n = 3 for each group. **(c)** Statistic analysis of the samples’ WCA.


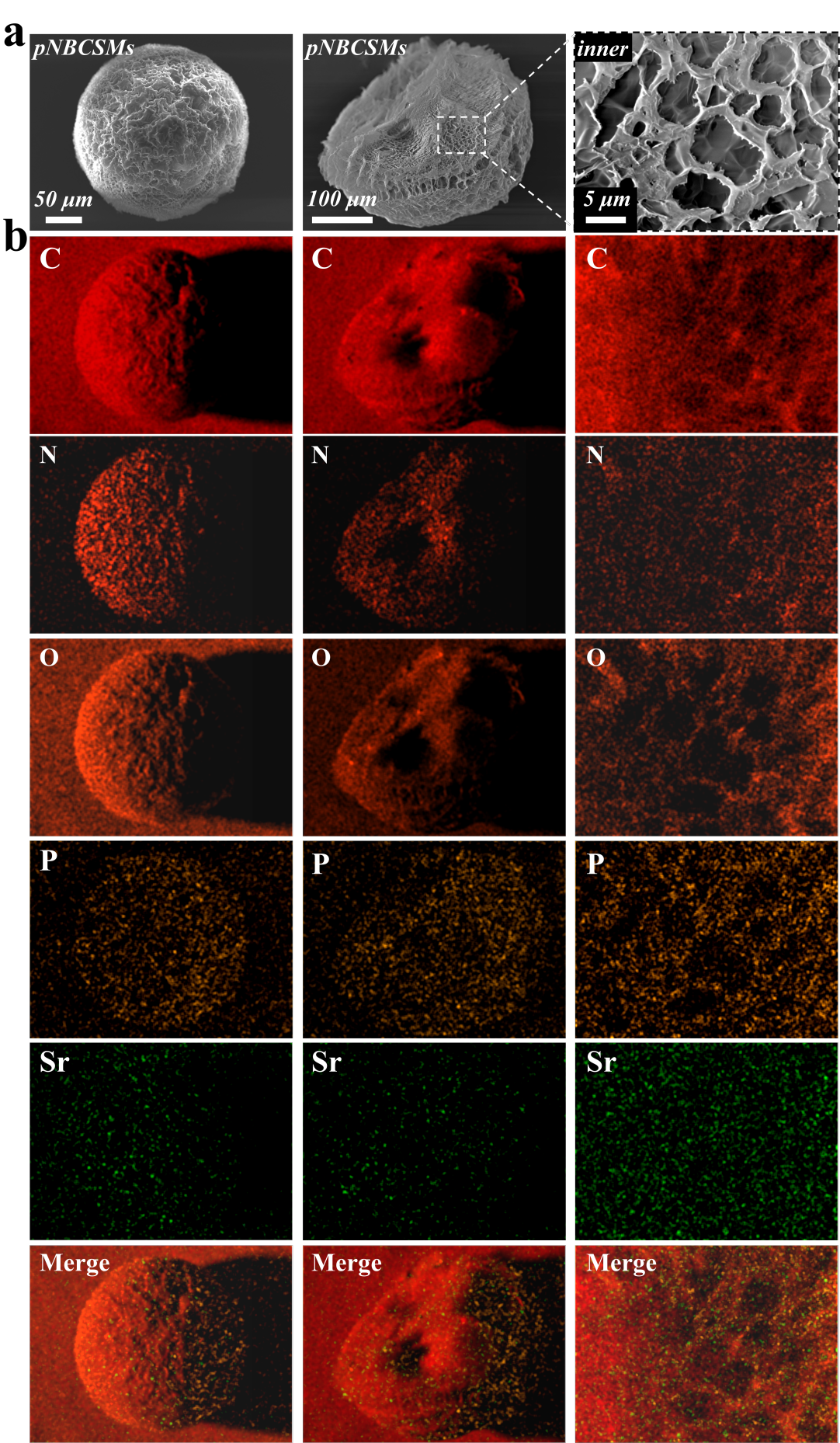


**Fig. S6 Characterization of the pNBCSMs** SEM **(a)** and EDS **(b)** images of pNBCSMs, wherein the first column represents a whole pNBCSM, the second column represents a half pNBCSM showing the inner surface, and the third column refers to the magnification of the inner surface.


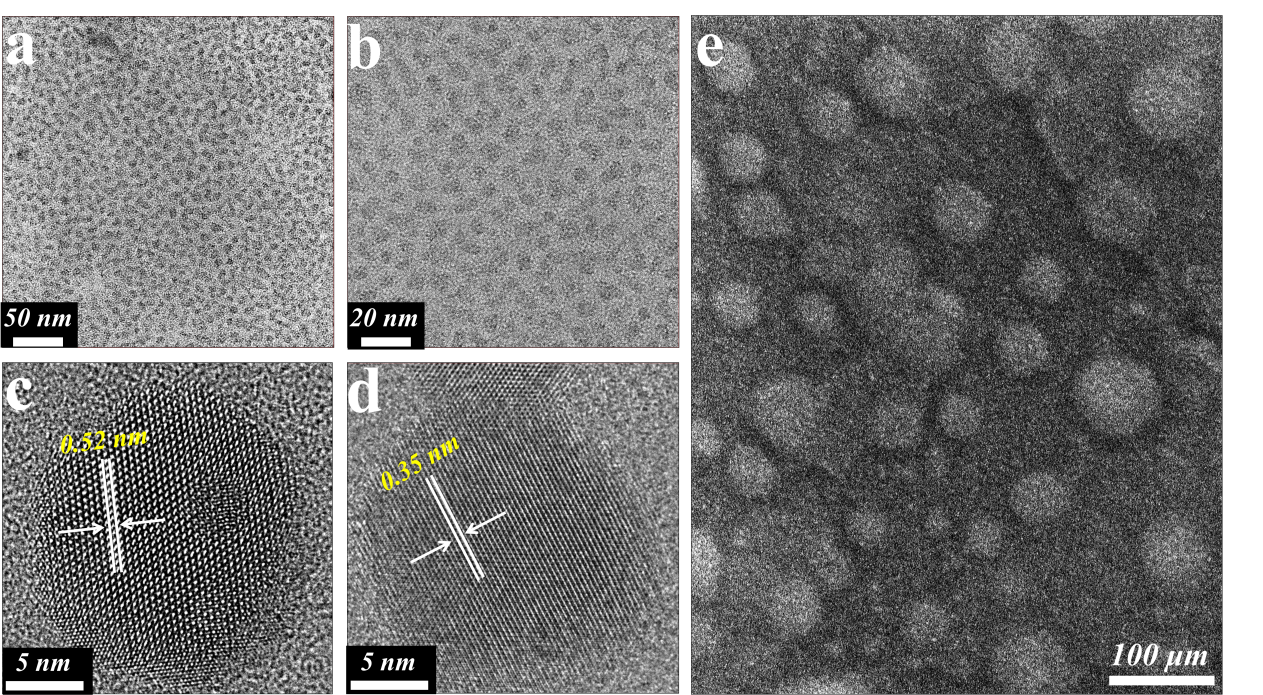


**Figure S7. Morphology characterization of black phosphorus (BP) (a)** TEM image of BP. **(b)** Enlarged TEM image of BP. **(c, d)** HRTEM images of BP with different lattice fringes. **(e)** SEM image of the BP.


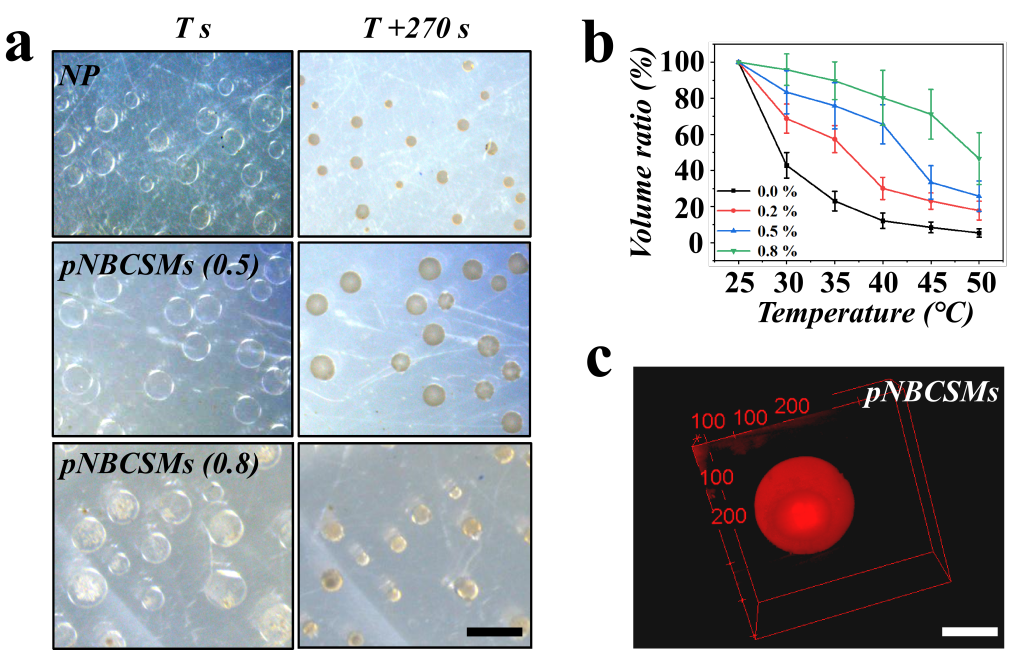


**Fig. S8 Characterization of the pNBCSMs (a)** The thermoresponsive behaviors of the pNBCSMs on the heating stage when the temperature was set to 40℃. The scale bar represents 400 µm. **(b)** The volume ratio of pNBCSMs with different CMCs concentrations at different temperatures (n = 3 for each group). **(c)** Three-dimensional images of pNBCSMs captured by confocal microscopy. The scale bar represents 100 μm.


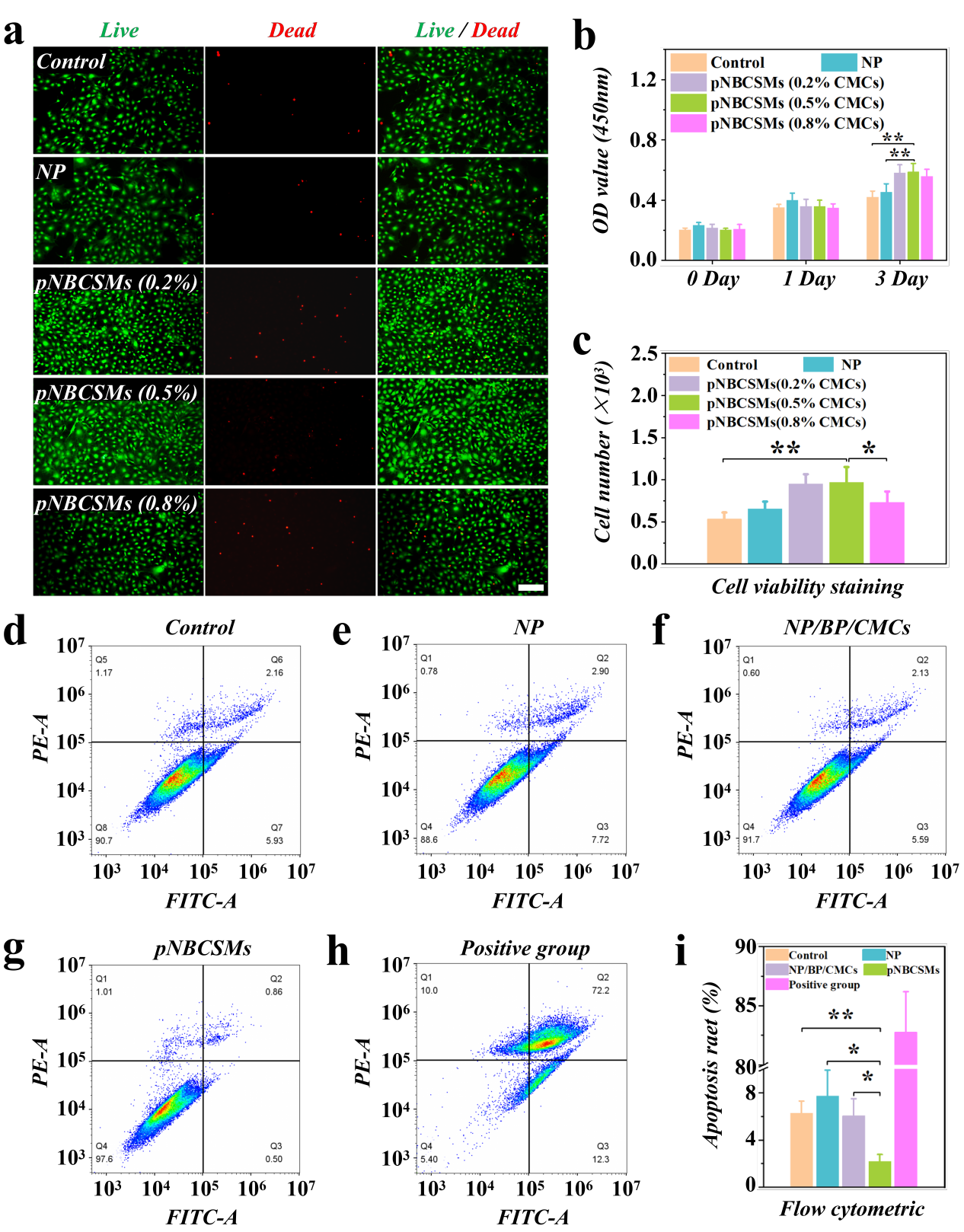


**Fig. S9** **Cell proliferation and apoptosis (a)** Live/dead staining images of MC3T3-E1 cells in each group. The scale bar represents 50 µm. **(b, c)** CCK8 result of MC3T3-E1 cells in each group (n = 5). **(d-h)** Apoptosis result of MC3T3-E1 cells detected by flow cytometry in each group (n=3). **(i)** Statistical analysis of apoptosis result of MC3T3-E1 cells.


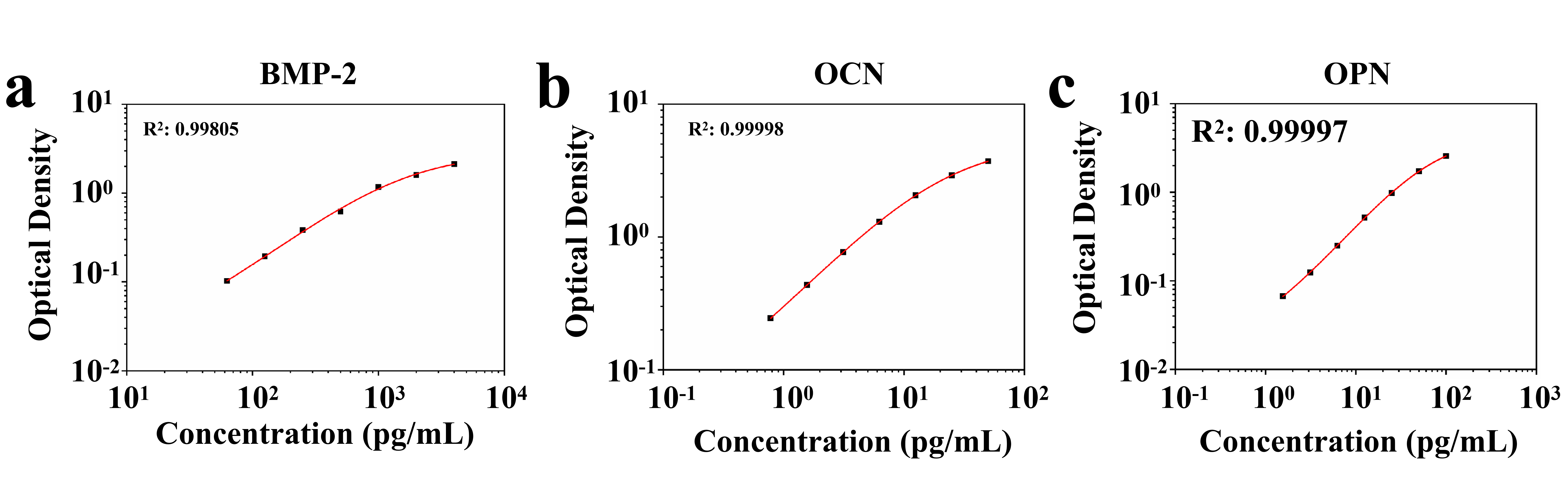


**Fig. S10** **Standard curves of osteogenesis-related proteins by ELISA kit**


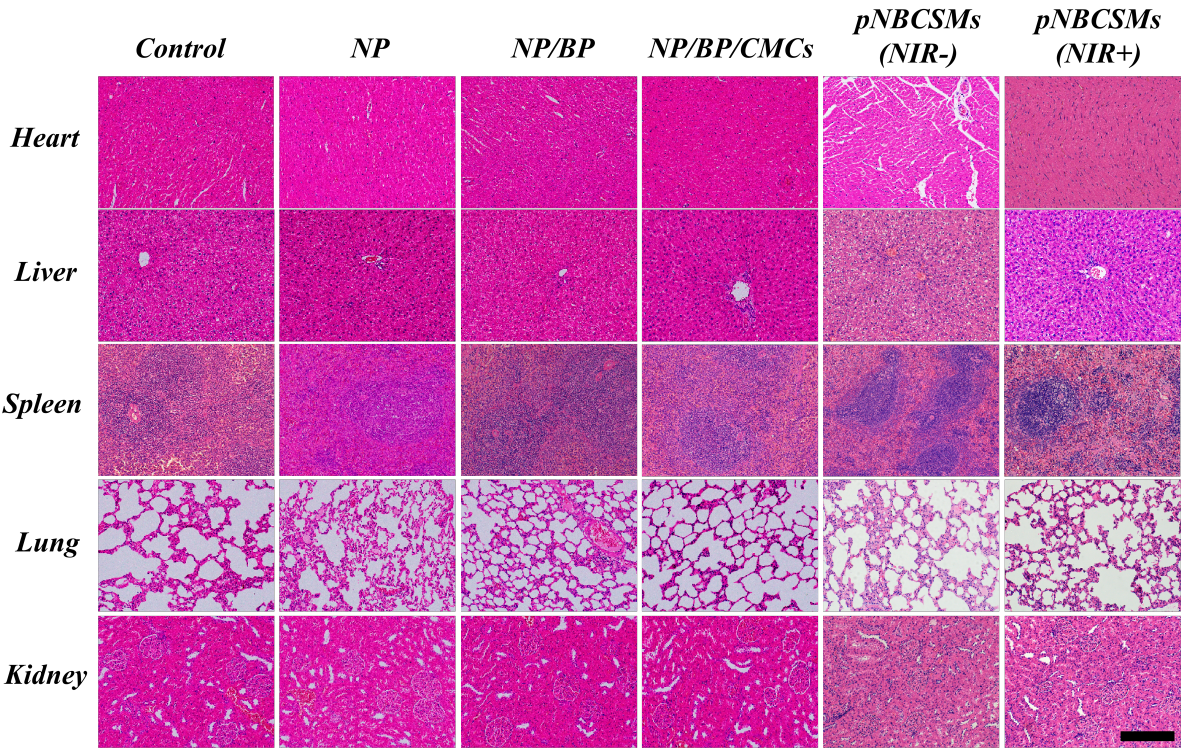


**Fig. S11 pNBCSMs has no toxic effect on tissues and organs in vivo** Representative H&E staining photographs of heart, liver, spleen, lung and kidney after exposure NP, NP/BP, NP/BP/CMCs, pNBCSMs (NIR-) and pNBCSMs (NIR+) to mice. The scale bar is 200 μm. n = 4 mice per group.
